# Effects of Slope Aspect and Rainfall on Belowground Deep Fine Root Traits and Aboveground Tree Height

**DOI:** 10.3389/fpls.2021.684468

**Published:** 2021-10-19

**Authors:** Ma Lihui, Liu Xiaoli, Chai Jie, Wang Youke, Yang Jingui

**Affiliations:** ^1^Institute of Water Saving Agriculture in Arid Areas of China, Northwest A&F University, Yangling, China; ^2^Institute of Soil and Water Conservation, Northwest A&F University, Yangling, China; ^3^College of Water Resources and Architectural Engineering, Northwest A&F University, Yangling, China; ^4^Language Culture College, Northwest A&F University, Yangling, China

**Keywords:** fine root, functional trait, root distribution, slope aspect, vertical tree extension

## Abstract

The vertical root distribution and rooting depth are the main belowground plant functional traits used to indicate drought resistance in arid and semiarid regions. The effects of the slope aspect on the aboveground traits are visible but not the belowground deep root traits. We aimed to investigate the fine root traits of the locust tree (*Robinia pseudoacacia* L.) planted on southerly and northerly aspects, and the variations in the rooting depth in regions with different rainfall, as well as assessing how deep rooting, might affect the response to drought in a loess region. We selected three study sites with different rainfall amounts, with six sampling plots at each site (three each with southerly and northerly aspects). Soil core samples were collected down to the depth where no roots were present. The locust trees tended to develop deeper fine roots rather than greater heights. The tree height and diameter were greater for locust trees on northerly aspects, whereas trees on southerly aspects had significantly deeper rooting depths. Fine root traits (root length, root area, and root dry weight density) were higher in the southerly aspect for both Changwu and Ansai, but lower in Suide. The ratio of the root front depth tree height ranged from 1.04 to 3.17, which was higher on southerly than northerly aspects, and it increased as the rainfall decreased. Locust tree growth traits (belowground fine root and aboveground tree height) were positively correlated with the mean annual rainfall. The soil moisture content of the topsoil decreased as the rainfall decreased, but the pattern varied in the deep layer. Our results suggest that the variations in the belowground rooting depth under different slope aspects may be related to plant survival strategies. The vertical extension of the rooting depth and tree height may be key functional traits that determine plant growth in drought-prone regions.

## Introduction

Determining plant functional traits might help us to understand the responses of plants to environmental variations (Reich et al., 2003). Solar radiation, temperature, and soil moisture are the main environmental factors that contribute to plant growth. The slope aspect is an important topographical factor because it can change the intensity and duration of solar radiation, thereby affecting the temperature and soil moisture in local areas (Cantón et al., 2004). The contrasting effects of northerly and southerly slopes can produce different climates, where the greater solar radiation on southerly slopes can increase the temperature and evapotranspiration to yield more arid conditions in the Northern Hemisphere (Finkel et al., 2001; Gutiérrez-Jurado et al., 2013). These climatic differences can cause plants to develop different functional traits, especially in terms of the tree height, canopy size, leaf structure, and branch density (Niinemets, 2006; Rozendaal et al., 2006). Finkel et al. (2001) and Nevo (2012) showed that the aboveground biomass was lower in a plantation on a southerly slope, whereas Kou et al. (2013) and Fan et al. (2012) obtained the opposite results. These differences of slope aspect and soil moisture in environmental factors could affect the aboveground morphological traits of plants (Vitória et al., 2019) and they might reflect the growth strategies employed by plants to resist drought (Liu and Ma, 2015). However, little information is available regarding the deep root traits of plants.

Fine roots (diameter < 2mm) play important roles in the absorption of water and nutrients (Chahine, 1992), especially the deep fine roots, which can absorb deep water in the soil to maintain plant transpiration during the dry season (Nepstad et al., 1994; Liu and Diamond, 2005; Oliveira et al., 2005). The ability of plants to access water reflects its adaptation to drought (Vadez, 2014). The fine root distribution and the rooting depth are the main belowground functional traits that might determine the volume of soil water acquired, thereby characterizing the growth and survival strategies of plants in water-limited ecosystems (Collins and Bras, 2007). Akkermans et al. (2012) showed that the rooting depth was a major variable that controls evapotranspiration during the dry season in African forests according to soil–vegetation–atmosphere transfer models, and it might be a direct indicator of a plant’s adaptability to drought. However, few studies have quantified the fine root distribution relative to rooting depth due to the difficulties of root sampling (Wang et al., 2009; Maeght et al., 2013; Freycon et al., 2014). In addition, studies have rarely investigated the deep fine root distributions under contrasting slope aspects (Kurze et al., 2017). Previous studies mainly concentrated on the differentiation of ecotypes on a large-scale aridity gradient (Wang and Gao, 2003; Volis, 2007; Kurze et al., 2017). According to the IPCC et al. (2013), the aridity will intensify in many regions throughout the world in the present century and the annual precipitation will decrease. Thus, deep fine root traits in regions with different rainfall amounts are valuable data for assessing hydrobiological processes and the adaptability of plants to environmental variations.

The Loess Plateau region of China is an ideal area for studying the root distributions in a deep and homogeneous soil layer because the deep loess layer is not affected by any interference due to differences in the groundwater level. The locust tree (*Robinia pseudoacacia* L.) is an exotic species that has been planted widely in China to conserve soil and water since the 1950s due to its drought tolerance, rapid growth, and simple propagation (Wang, 2017). The area planted with locust trees since the 1950s exceeds 70,000ha in the arid and semiarid Loess Plateau region (Guo et al., 1998). The locust tree is characterized by high water consumption and it can readily exhaust the available soil water. However, it is highly resistant to drought when the minimum survival requirement is maintained. Zhang et al. (2018) found that the fine root area density in locusts tree exhibited a decreasing trend from a wetter site to a drier site, and the fine roots exhibited similar rooting patterns along a precipitation gradient throughout the whole 3m soil profile. Nicolescu et al. (2018) showed that the locust tree was fast growing with the maximum tree height being reached in the first 5years and the peak diameter in the first 10years. The locust tree has a high capacity for adaptability and it can be utilized in projects that aim to convert farmland into forestland, it can severely desiccate the deep soil layer (Jia et al., 2017), thereby resulting in environmental deterioration with low productivity and efficiency.

In the present study, we comprehensively analyzed the vertical extent comprising the tree height and the rooting depth for the locust tree. We hypothesized that the rooting depth would increase as the rainfall decreased and it was deeper in northerly than southerly aspect according to the theory of optimal resource partition which indicated that plant root could extend deeper layer to absorb deeper soil water to resist drought in drier areas. The main aims of this study were as: (1) to investigate the deep fine root distribution for locust trees under different slope aspects; (2) to analyze the trends in the fine roots in regions with different rainfall amounts; and (3) to assess the vertical extents of the rooting depth and tree height as key functional traits for determining plant growth. The results obtained in this study provide new insights into the vertical extent of the deep roots and the ratio of the belowground rooting depth relative to the aboveground tree height in areas with different rainfall amounts, thereby helping us to understand plant growth and survival strategies.

## Materials and Methods

### Study Sites

**Figure 1:**
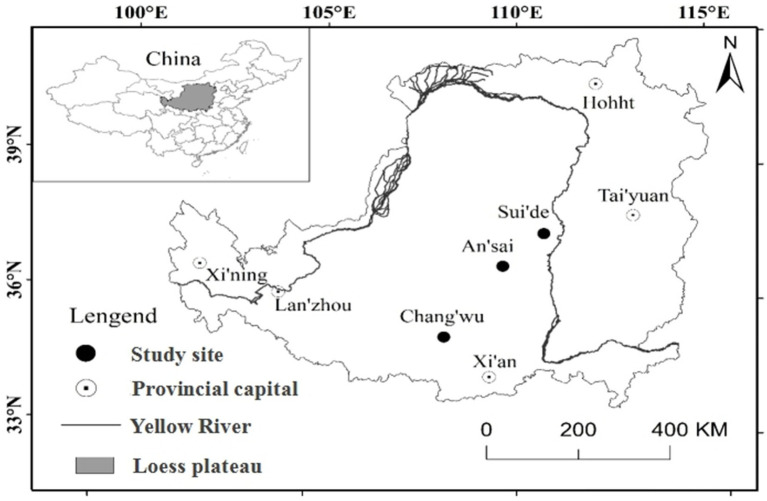
Study sites for locust tree in loess region.

This study was conducted during summer between July and August 2018 in the Loess Plateau region of China, where the deep loess layer and ground water depth are more than 50m (Ma et al., 2013; Zhu et al., 2018). Thus, this region is an excellent area for studying deep roots. In our study area, we investigated three different sites in Changwu, Ansai, and Suide counties across a distance of 337km. In this region, the climate is cold with four distinct seasons characterized by dry winters and warm summers in Changwu and Ansai (Dwb), and the arid conditions characterized by steppe and cold (Bsk) in Suide according to the Köppen-Geiger climate type (Peel et al., 2007). The highest sunlight, heat, and rainfall levels mostly occur in July, August, and September, and more than 50% of the total annual rainfall occur in these 3months (Table 1). The locations of the sites are shown in Figure 1. We sampled the northerly and southerly aspects at all sites with three samples from each.

**Table 1:** Main natural geographical characteristic and soil properties at sampling sites.

Site	Annual rainfall (mm)	Annual temperature (°C)	Altitude (m)	Annual sunshine hours (h)	Annual potential evapotranspiration (mm)	Cumulative temperature (≥ 10°C)	Soil bulk density (gcm^−3^)	Soil field capacity (%)	Soil wilting point (%)	Climate type	Geographical coordinates
Changwu	580	9.1	1,224	2226.5	1029.1	3,029	1.3	21.9	7.46	Cold with dry winter and warm summer (Dwb)	107°48'–107°58'E, 34°59'–35°18'N
Ansai	500	8.8	1,063	2395.6	1,127	3,121	1.28	18.4	4.5	Cold with dry winter and warm summer (Dwb)	108°05'–109°26'E, 36°30'–37°19'N
Suide	443	9.7	877	2615.1	1280.5	3,485	1.25	15.8	3.7	Arid with steppe and cold (Bsk)	110°04'–110°41'E 37°16'–37°45'N

*Soil bulk density, soil field capacity, and soil wilting point were measured in 0–1m layer. Climate was based on updated Köeppen-Geiger climate classification* (Peel et al., 2007).

At each site, we selected six sampling plots on the upper slopes (10×10m, with three on southerly aspects and three on northerly aspects) to quantitatively investigate the characteristics of the locust tree plantations. The sites were chosen carefully to minimize confounding environmental factors, where the altitude, inclination, and tree growth stage were similar on both aspects (three replicates). All sites were located in well-vegetated areas where the depth of the surface litter was about 1cm in the locust tree stands. The sampling point was located on the same side of the tree along the contour line. The sampling point and two adjacent locust trees (2m between the trees and 4m between the tree rows) at a similar growth stage in the same row formed an isosceles triangle with a side length of 1.2m from the tree trunk. We transformed the slope into flat ground with a shovel and fixed sampling shelves with two wooden baseboards (1.2m in length and 0.3m in width), where a plumb was used to determine the level. All weeds and the litter layer were removed every 10days at 30, 20, and 10days before sampling period within the sampling area to eliminate any interference from other plant roots.

The maximum rainfall infiltration depth was 2m (Liu et al., 2010). We defined the deep soil as the soil layer below 2m. Liu et al. (2014) showed that the deep soil moisture content (below 2m) remained fairly stable with almost no seasonal variation based on long-term observations in the study area. Thus, we assumed that the deep soil moisture was not affected by the rainfall in the sampling period and it could represent the soil moisture regime consumed by plants over a long period. According to previous studies, the sampling period during the summer is characterized by the peak abundance of fine roots (Burke and Raynal, 1994), minimum soil water storage (Duan et al., 2017), and the most severe drought throughout the year. Therefore, the data collected represented the responses of the fine roots to the soil water regime and other environmental factors in the corresponding sampling period.

### Stand Characteristics

The locust tree stands at the three sites had similar densities (5,000 trees ha^−1^), with 2m between the trees and 4m between the tree rows, and the canopy was closed with coverage of more than 85%, except on the southerly aspect in Ansai where the coverage was 70%. The stands were all artificial forests, which were in middle age in Ansai (18years) and mature age in Changwu and Suide (37 and 35years) (Table 2); the trees were all at the growth period based on the regulations for the main tree species (LY/T 2908–2017, China), which define the age of locust trees as young at ≤15years, middle at 16–25years, and mature at 31–40years. The tree height and mean diameter at breast height were determined. We measured the tree height with an optical height meter (CGQ-1, Harbin Optical Instrument Factory Co. Ltd., China) and the mean diameter with a measuring tape. First, we measured the horizontal distance between the trees and the optical height meter with a measuring tape. We then targeted the top of the tree through an observation hole and determined the sight point carefully by aligning the pointer in the plumb position. We pulled the trigger and the pointer was locked. Hence, tree height was determined by the pointer scale values. The coverage represents the percentage of the vertical projection area of the plant aboveground relative to the sampling plot area, and it was calculated using the formula for the elliptical projected area of the canopy: C=πXY/4 , where X and Y denote the major and minor axis lengths, respectively (Phillips and Macmahon, 1981).

**Table 2:** Main characteristics of locust tree stands.

Location	Slope aspect	Slope gradient	Age (years)	Annual cumulative PAR (molm^−2^ s^−1^)	Maximum daily temperature	Stand height (m)	Diameter at breast height (cm)	The depth of the root front (m)	Ratio between the depth of root front and stand height	Whole vertical extension (m)	Coverage	Main understory vegetation
Changwu	North	37°	37	9,320	32.2°C	10.03 ± 1.73 a	10.53 ± 2.70 ab	10.4 ± 0.33DE	1.04	20.4	96%	*Rubus*
	South	36°	37	9,861	33.4°C	7.45 ± 1.57 bc	9.4 ± 2.56 abc	18 ± 0.78 A	2.42	25.5	98%	*Leymus*
Ansai	North	33°	18	9,440	34.5°C	7.75 ± 0.70 b	12.05 ± 2.78 a	11.8 ± 0.21 CD	1.52	19.6	95%	*Armoise*
	South	31°	18	9,988	37.1°C	6.59 ± 1.77 bc	6.89 ± 1.89 bc	14 ± 0.65 B	2.12	20.6	70%	*Agropyron*
Suide	North	29°	35	9,510	34.2°C	5.20 ± 0.79cd	7.53 ± 1.51 bc	9.6 ± 0.96 E	1.85	14.8	85%	Moss
	South	32°	35	10,062	36.8°C	4.04 ± 0.28 d	5.30 ± 1.41 c	12.8 ± 0.14BC	3.17	16.8	85%	*Armoise*

*Different letters for stand height, diameter, and the depth of the root front indicate significant differences among sampling sites on different slope aspects (n=3, p < 0.05)*.

*PAR indicates photosynthetically active radiation. Whole vertical extension refers to the stand height plus the maximum rooting depth. Coverage represents the percentage of the vertical projection area of the plant above ground relative to the sampling plot area*.

### Fine Root Sampling and Analysis

Fine roots were sampled using the soil coring method (with a specialized soil auger made in China called a “Luoyang shovel” internal diameter=16cm), as described previously by Ma et al. (2013). In fact, a massive effort was done to achieve such work through very deep soil excavation. Luoyang shovel with larger internal diameter could greatly improve the sampling efficiency, it took about 2.5h for two persons to drill down to 8m depth, 4h for two persons to 12m depth, and 8h for three persons to 18m depth. Soil samples were collected at 20cm intervals throughout the whole soil layer until no roots were found in two consecutive layers. We set the “two consecutive layers” criterion for root front depth which meant that root front extended downward to the depth where no root occurred below this depth in two consecutive layers. Deep roots were defined as roots found below 2m. The vertical tree extension was defined as root front depth plus tree height.

Roots were separated from soil particles by washing through a 0.2mm sieve under running tap water. The root samples were placed in plastic bags, tagged with identifiers, and stored at 4°C until they were processed. Tree roots were distinguished from grass roots based on their morphology and color (Sun et al., 2015). Dead roots were identified based on their color and flexibility, and then discarded (live roots were yellowish brown, whereas dead roots were dark). We found a few dead roots in the top 1.2m soil depth and almost no decay roots occurred in deeper soil layer. Fine roots (diameter < 2mm) were collected and placed on transparent rectangular plastic plates filled with water, which allowed the roots to spread and float on the surface, before scanning at a resolution of 300 dpi. The area, length, and diameter were measured for the fine roots with WinRHIZO (Regent Instruments Inc., Canada). The fine root length density (RLD, m m^−3^) was defined as the root length per unit soil volume. D_50_ and D_95_ were used to denote the soil depths above which 50% or 95% of the total RLD were located, respectively. The fine root area density (RAD, m^2^ m^−3^) was defined as the root area per unit soil volume. The fine root dry weight density (RDWD, g m^−3^) was defined as the dry weight of roots per unit soil volume, which was determined after washing and cleaning, and drying for 24h at 65°C. Nothing was present in the deep layer except for soil and roots. We found only a few roots above 1cm in diameter below 3m. The eolian soil was homogeneous among the six sampling points at each site in the loess region. At present, the soil coring method is a feasible approach for identifying deep roots by actual field sampling.

### Soil Moisture Analyses

Soil bulk density, soil field capacity, and soil wilting point were measured in 0–1m layer in the laboratory. Soil bulk density and soil filed capacity were determined by the cutting ring method proposed by Zhong and Zhang (2014). The wilting point was measured following the method described by Wei et al. (2004), who defined the wilting point as the soil water content when the soil water suction was equal to 1.47MPa.

After separating the roots, each soil core sample was dried to a constant weight at 105°C and the gravimetric soil moisture content was determined using the following formula.


$$\mathrm{soil moisture}\;\left(\%\right)=\frac{\mathrm{wet}\;\mathrm{soil weight}-\mathrm{dry}\;\mathrm{soil weight}}{\mathrm{wet}\;\mathrm{soil weight}}\times 100\%$$

Soil organic carbon, soil total nitrogen, and soil total phosphorus concentration were measured on the southerly and northerly aspect in the whole sampling soil depth in Changwu (unpublished data); we found that soil nutrients were concentrated on surface layer (0–40cm) and varied little with soil depth. Soil organic carbon content was larger on northerly than southerly aspect, while the total nitrogen and phosphorus content kept fairly consistent on different slope aspect. Soil organic carbon, total nitrogen, and phosphorus content were largest in Changwu, while they remained roughly similar values in Ansai and Suide (Zhang et al., 2018).

### Statistical Analysis

A general linear model and multi-way analysis of variance were used to test the effects of the soil depth, slope aspect, site, and the interactions between the slope aspect × site, slope aspect × soil depth, site × soil depth, and slope aspect × soil depth × site on the fine root diameter, RLD, RAD, and RDWD. The slope aspect and site were treated as fixed effects, and the soil depth as a continuous variable and covariate. F-tests were conducted to assess the significance of the site, slope aspect, and their interaction. We log-transformed the root biomass values to satisfy the requirements for homoscedasticity and normality. All analyses were conducted with SPSS 11.0 (SPSS Inc., Chicago, Illinois, United States). Duncan’s test was used to determine significant differences (*p* < 0.05).

## Results

### Fine Root Traits

**Figure 2:**
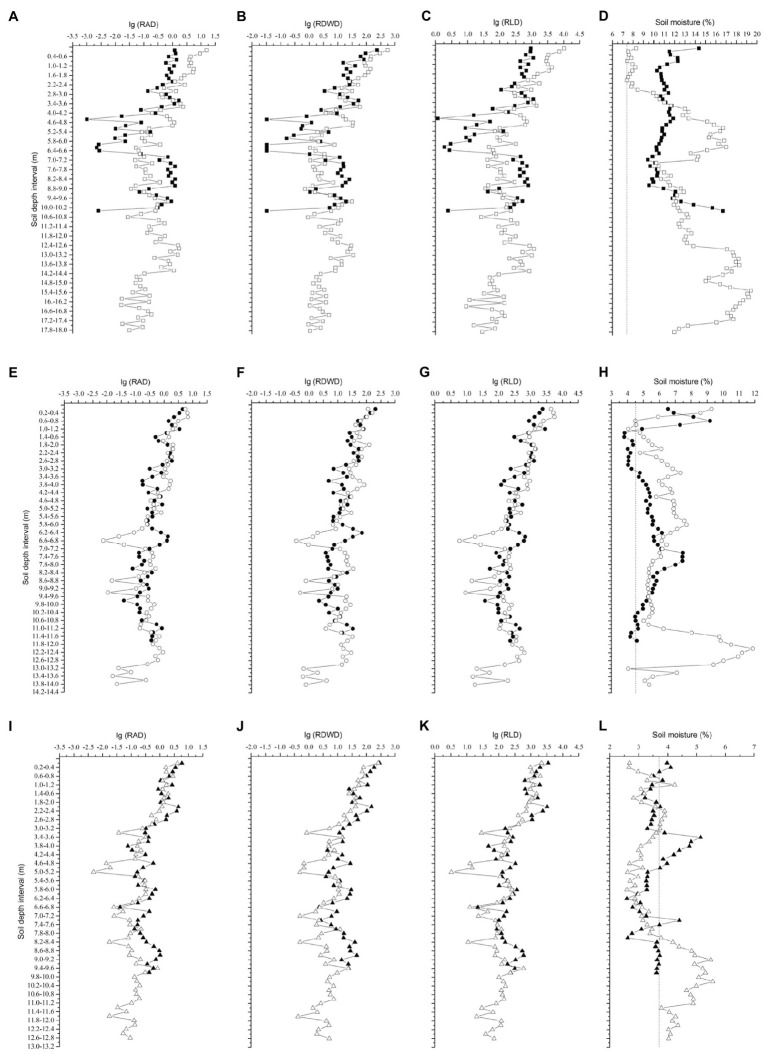
Changes in RAD, RDWD, RLD, and soil moisture down to the root front with soil depth. RAD is the root area density (m^2^ m^−3^), RDWD is the root dry weight density (gm^−3^), and RLD is the root length density (mm^−3^). Dashed lines denote the wilting point at each site. Solid and empty rectangles, circles, and triangles represent northerly and southerly aspects at the sites Changwu **(A,B,C,D)**, Ansai **(E,F,G,H)**, and Suide **(I,J,K,L)**, respectively.

**Figure 3:**
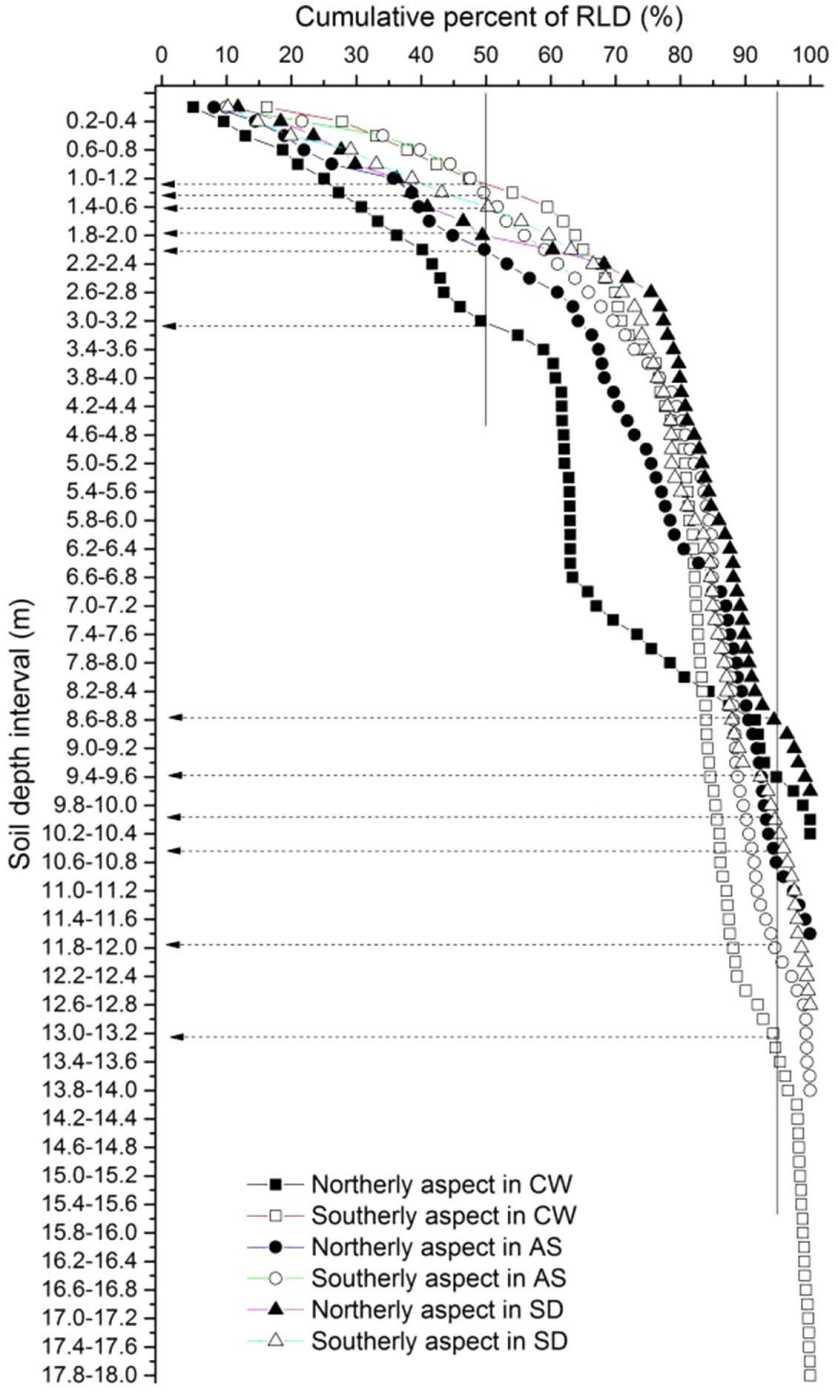
Cumulative percentages of fine root length density down to the root front with soil depth. Solid lines denote the 50 and 95% total root length densities. Dashed lines are the corresponding soil depths above which 50 and 95% of the total root length densities are located. Solid and empty rectangles, circles, and triangles represent northerly and southerly aspects at the sites Changwu, Ansai, and Suide, respectively.

The fine roots of the locust trees exhibited similar trends, where RLD, RAD, and RDWD all decreased sharply in the top 2.6m of the soil, varied slightly from 2.6–5m, and then remained stable below 5m (Figure 2). The maximum RLD and RAD occurred in the top 0.2m on the northerly aspect in Ansai, and the maximum RDWD on the southerly aspect in Changwu. More than 50% of the total RLD was concentrated in the upper 2.2m (except for 3.4m on the northerly aspect in Changwu) and less than 5% below 8.6m (Figure 3).

We found that trees on southerly aspects had higher RLD, RAD, and RDWD values, except for Suide (Table 3). The total RLD, RAD, and RDWD values decreased as the rainfall decreased on southerly aspects. However, the total RAD and RDWD increased on northerly aspects, while the total RLD values were similar in Ansai and Suide, and greater than those in Changwu. Though fine root traits (RLD, RAD, and RDWD) decreased with the rainfall amount, there were no significant differences among the three sites (Table 4). The effects of the soil depth on RLD, RAD, and RDWD were highly significant (*p* < 0.01). The slope aspect had significantly different effects on RLD (*p* < 0.01) and RAD (*p* < 0.05) but not on RDWD. The mean root diameter varied little and it tended to be uniform throughout the whole soil depth (Table 3).

**Table 3:** Main characteristics of fine roots on different slope aspects.

Location	Slope aspect	Total root length density (mm^−3^)	Soil layer where the cumulative percentage of total root length density occurs (m) D_50_ D_95_	Total root area density (m^2^ m^−3^)	Total root dry weight density (gm^−3^)	Mean diameter (mm)
Changwu	North	19397.2 ± 190.2 e	3.4 9.8	28.3 ± 0.2 d	1032.6 ± 27.9 f	0.45 ± 0.08 a
	South	62967.3 ± 611.3 a	1.4 13.6	90.7 ± 7.8 a	2493.0 ± 69.6 a	0.43 ± 0.08 a
Ansai	North	29688.3 ± 277.5 c	2.2 10.8	45.5 ± 1.2 c	1541.9 ± 41.9 d	0.47 ± 0.08 a
	South	44395.9 ± 414.9 b	1.6 12.2	62.9 ± 8.9 b	2005.9 ± 54.4 b	0.48 ± 0.08 a
Suide	North	29651.1 ± 270.3 c	2.2 8.6	47.7 ± 0.2 c	1898.0 ± 50.7 c	0.54 ± 0.09 a
	South	21161.9 ± 201.5 d	1.6 10.2	30.3 ± 5.1 d	1121.4 ± 30.7 e	0.42 ± 0.07 a

*Different lowercase letters for total root length density, total root area density, total root dry weight density, and mean diameter indicate significant differences among sampling sites on different slope aspects (n=3, p < 0.05)*.

**Table 4:** F-values for the effects of site, slope aspect, and depth, and the interactions between site × slope aspect, site × depth, slope aspect × depth, and site × slope aspect × depth based on fine root length density (RLD, m m^−3^), fine root area density (RAD, m^2^ m^−3^), fine root dry weight density (RDWD. g m^−3^), and fine root diameter (mm).

	RLD	RAD	RDWD	Diameter
Site	F2,372=0.901	F2,372=0.532	F2,372=0.351	F2,372=3.316
Slope aspect	F1,372=7.934^**^	F1,372=5.886^*^	F1,372=0.006	F1,372=0.759
Depth	F1,372=92.038^**^	F1,372=92.602^**^	F1,372=102.883^**^	F1,372=2.223
Site × Slope aspect	F2,372=12.060^**^	F2,372=13.987^**^	F2,372=6.441^**^	F2,372=0.649
Site × Depth	F2,372=1.134	F2,372=1.729	F2,372=1.291	F2,372=1.119
Slope aspect × Depth	F1,372=0.702	F1,372=0.460	F1,372=2.116	F1,372=18.109^**^
Site × Slope aspect × Depth	F2,372=4.912^**^	F2,372=5.758^**^	F2,372=2.013	F2,372=8.009^**^

* *Significant difference at p < 0.05*;

** *significant difference at p < 0.0*.

### Vertical Tree Extension

**Figure 4:**
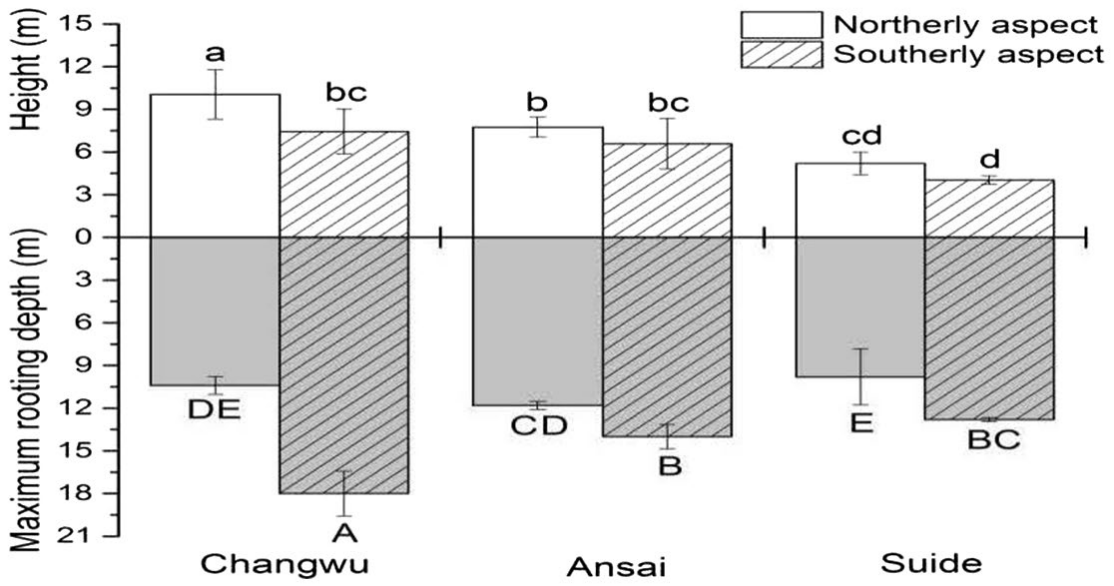
Mean stand heights and the depth of the root front at Changwu, Ansai, and Suide. Vertical bars indicate standard deviations. Lowercase letters indicate significant differences in stand height and uppercase letters indicate significant differences in the depth of the root front (n=3, *p* < 0.05).

Our results showed that the vertical extents of the trees varied among sites and slope aspect (Figure 4; Table 2). The tree height and diameter at breast height decreased as the rainfall amount decreased, and they were also higher on northerly than southerly aspects (Table 2). The mean root front depth also decreased as the rainfall amount decreased, but it was much deeper on southerly than northerly aspects in the same site, where the difference was significant. Root front depth ranged from 9.6m on the northerly aspect in Suide to 18m on the southerly aspect in Changwu. The heights of the locust trees tended to be shorter than the depths of the root front. The ratio between the belowground root front depth and aboveground tree height ranged from 1.04 to 3.17, which was higher on southerly than northerly aspects, and it increased as the rainfall amount decreased. The vertical tree extension values were also larger on southerly than northerly aspects but they decreased as the rainfall amount decreased (Table 2).

### Soil Moisture

The mean soil moisture contents in the 0–2m layer were higher on northerly than southerly aspects at each site, which were 11.55 and 7.8% in Changwu, 5.93 and 5.75% in Ansai, and 3.6 and 3.15% in Suide (lower than the wilting point of 3.7%), respectively. However, the tendency of mean soil moisture contents from 2m to the root front depth was opposite, which were 11.32 and 14.1% in Changwu, 5.34 and 6.65% in Ansai, and 3.57 and 3.83% in Suide, respectively. We found that the mean soil moisture contents of the top soil layer were higher on northerly than southerly aspects at each site and they decreased as the rainfall decreased. The soil moisture contents varied below 2m (Figures 2D,H,L).

## Discussion

### Deep Root Traits

Our results indicated that the mean RLD, RAD, and RDWD decreased as the rainfall amount decreased. Locust trees on southerly aspects had higher root biomasses, but the opposite was found in Suide, possibly because the lower root diameter and weaker growth led to smaller biomasses (Table 3) due to severe soil moisture depletion lower than wilting point. We found that the total RLD on southerly aspects was approximately three times higher in Changwu compared with that in Suide (Table 3; 62967.3mm^−3^ vs. 21161.9m m^−3^). A previous study also showed that the root biomass was four times higher in mesic populations (Kurze et al., 2017). Volis et al. (2002) and Mendola et al. (2015) determined a similar trend to our results for an annual grass. There may be intensified plant–plant competition for belowground water and nutrient resources in mesic environments (Metz and Tielbőrger, 2016). If the competition supersedes the effect of regional climate on the availability of belowground resource, i.e., less resources are available per individual in mesic sits, then the allocation of resources to the roots will be higher in a mesic environment compared with an arid environment (Kurze et al., 2017). However, these results were opposite to those predicted by optimal resource partitioning, where the investment in roots was expected to be higher under lower soil water conditions (Bloom et al., 1985). Aronson et al. (1992) also found that the allocation to roots was higher in an arid population during the early life stage. While this trend gradually reversed with age, thereby indicating that the maturing plant allocated more biomass to seed production rather than roots which might reflect a strategy for maximizing reproductive production at the expense of vegetative growth.

Pinheiro et al. (2016) found that the fine root densities decreased in the topsoil but remained roughly similar at depths of 0.25 and 6m. We also found that RLD, RDWD, and RAD decreased sharply in the top soil, but they remained stable below 5m (Figure 2). Schenk and Jackson (2002a) showed that D_50_ and D_95_ were located in the upper 0.3m and 2m, respectively. However, we found that D_50_ and D_95_ moved down to greater depths, especially D_95_, according to the experimental field measurements. The cumulative percentage RLD did not differ greatly in the shallow layers between northerly and southerly aspects, where the difference was mainly due to roots extending deeper on southerly aspects (Figure 3). Christina et al. (2011) and Laclau et al. (2013) showed that less than 5% of the fine root biomass of *Eucalyptus grandis* seedlings was present below a depth of 8m. We obtained similar results where less than 5% of the fine RLD was located below 8.6m (Figure 3). Christina (2015) reported that very low densities of the fine roots of *E. grandis* below a depth of 10m could meet most of the water requirements during dry periods after canopy closure. Indeed, an extremely low deep fine root density might contribute to improving tree survival in extremely arid regions. We speculated that variability in the rooting depth might be related to the survival of trees on different slope aspects in arid regions.

### Soil Moisture

Plants can maintain a balance between the supply and demand of water by regulating the proportions of the aboveground and belowground plant parts (Tian et al., 2014). Tian et al. (2014) reported that the root: shoot ratio increased when *Haloxylon* seedlings suffered from severe water stress and a new water balance mechanism formed, which facilitated survival under soil moisture contents below 1.5%. We found that root front depth and root biomass (RLD, RAD, and RDWD) decreased as rainfall amount decreased and rainfall amount was positively related to soil moisture in the whole soil layer in three sampling sites. Severe soil moisture depletion up to the wilting point of 3.7% in Suide led to slow and stagnant growth, where the locust trees tended to be smaller and older, probably because of the rapid growth and high consumption of soil water in the early stage, and soil moisture could not meet the water requirements and environmental deterioration occurred accompanied by soil dry layer and degraded forest.

The infiltration of rainfall is quite complex and it is primarily influenced by factors, such as the soil texture, soil pore space, soil surface feature, slope position, and slope gradient. Ribolzi et al. (2011) found that the infiltration depth increased as the slope gradient increased. However, Jiang and Huang (1984) showed that the infiltration of rainfall decreased as the slope gradient increased due to the gradual decrease in the infiltration rate and increased runoff. Thus, the soil moisture content was higher on gentle slopes than steep slopes. In our study, we selected similar slope positions and soil surface feature to avoid the influence of rainfall infiltration. The soil moisture is an important factor for root growth (Zhou and Shangguan, 2007). We found that the mean soil moisture content in the 0–2m depth was higher on northerly than southerly aspects at each site (Figures 2D,H,L), possibly due to the greater photosynthetically active radiation and higher evapotranspiration on southerly compared with northerly aspects. Our results are consistent with those reported by Deng and Zhang (2010), whereas Sun et al. (2007) obtained the opposite results in the 1m surface layer. Wang (2017) found that the soil moisture content was higher in the topsoil and the tree height was lower on a shady slope compared with a sunny slope, where the main factor related to tree growth was soil aeration rather than the water content. We concluded that the soil moisture in the topsoil might have been affected by environmental factors, such as rainfall and soil transpiration, but the soil moisture content in the deep soil may have reflected differences of long-term rainfall in the soil and deep root characteristics, where an alternating pattern was detected at different sites (Figures 2D,H,L).

### Effects of Slope Aspect on Tree Height and Rooting Depth

In contrast to our hypothesis, we found that the effect of the slope aspect was greater on the belowground plant parts than the aboveground parts (Figure 4). The locust trees had greater rooting depths and smaller tree heights on southerly aspects than northerly aspects, possibly because the southerly aspects could receive more solar radiation, thereby leading to greater transpiration and lower relative humidity, and the available soil moisture by locust trees in surface soil layer was less, thus exhibited smaller tree height. As we all know, the aboveground growth depended on the water taken up by the roots (Salmerón-Miranda et al., 2007), we found that root extended deeper in southerly than northerly aspect, which showed that root could absorb deeper water from deeper soil layer to sustain plant growth, while it did not indicate a lower water moisture content because the mean deep soil moisture content was calculated from 2m to the root front depth., The reduced root in northerly aspect may reflect the strategy of belowground part to maximize reproductive allocation (Kurze et al., 2017). Thus, the locust trees appear to have adapted to the different water conditions caused by the different slope aspects, where they exhibited diverse survival strategies (vertical tree extension, i.e., tree height plus rooting depth) to cope with drought. Petrů et al. (2006) obtained similar results and found that the height of *Biscutella* was lower in arid populations. May et al. (2013) and Šímová et al. (2015) also found that the rooting depth differed greatly between northerly and southerly aspects, where the tree height varied along a regional rainfall gradient. However, Christina et al. (2011) showed that the vertical tree extents above and below ground were almost the same in *Eucalyptus* plantations in Brazil. Kurze et al. (2017) suggested that the relative investment in the root: shoot ratio was higher in mesic populations. However, we found that the ratio of the rooting depth relative to the tree height increased as the rainfall amount decreased, thereby indicating that the locust trees allocated more biomass to the belowground parts to cope with drought conditions.

Larson and Funk (2016) suggested that the rooting depth may be the key factor for differentiating the performance of plants under drought conditions. However, few previous studies have determined the correlation between the rooting depth and the mean annual rainfall. Bhattachan et al. (2012) reported that there was no obvious relationship between the rooting depth and mean annual precipitation in water-limited ecosystem. Zhou et al. (2020) found that the potential rooting depth was negatively correlated with the mean annual rainfall across Southern African savannas, and they suggested that the root strategies were highly diverse in more arid sites. They estimated that the rooting depth was 4.58m for drought-resistant *Birrea* based on an exponential decay function. However, we determined deeper rooting depths by conducting full vertical excavations and based on actual measurements. Contrary to our hypothesis, we found that the rooting depth and vertical tree extension all decreased as the rainfall amount decreased (Figure 4). Zou (1986) showed that annual rainfall of 550mm was the boundary for the vegetation type distribution from forest steppe to forest vegetation. Our experimental areas had annual rainfall amounts ranging from 443 to 580mm (Table 1). Thus, the difference in the annual rainfall combined with the evapotranspiration produced by the slope aspect could have affected plant growth, thereby reflecting the plant survival strategy. The locust trees were relatively vigorous in terms of both their aboveground and belowground parts in the areas with more rainfall, but not in arid areas because the low rainfall and poor water supply led to weaker growth. We considered that the rooting depth could be used as a single indicator to represent a plant’s survival strategy, and the tree height combined with the rooting depth as two criteria to understand the strategy employed by a plant to adapt to drought stress.

The changes in the rooting pattern with stand age might vary according to the root adaptation strategies relative to the availability of water and nutrients. Zhang et al. (2018) obtained a curve with a single peak for the roots as the trees aged, and the coefficient of the rooting distribution to stand age (β) peaked in 18-year-old locust trees (*β*=0.9912) with a greater rooting depth but then decreased sharply in 35-year-old stands (*β*=0.9847) with a shallower rooting depth. Chang et al. (2012) found that the rooting patterns were similar for 8- and 30-year-old locust stands in the Loess Plateau region. However, we could only investigate 18-year-old locust trees in the area sampled in Ansai. In addition, we found that the 18-year-old locust trees in Ansai were in a vigorous growth stage, whereas the trees aged over 35years were in a stage of decline in Suide and Changwu (Table 2). According to a study by Zhang et al. (2018), the rooting depth might have become shallower with age in Ansai, and it would be greater in Changwu than Ansai at the same stand age. The difference between Ansai and Suide means that it is also necessary to compare tree samples with similar ages.

The rooting depth and biomass allocation are important belowground functional traits (Liu and Ma, 2015). Our deep sampling procedure was laborious and time consuming, so we only sampled three trees on each slope aspect per site. The sample size was limited but given the valuable quantitative data obtained, we consider that sampling three trees may have been representative of the actual root distributions. We found only a few roots with lengths of about 1cm below 2m in the soil cores, and our previous study also showed that that deep root biomass varied little with the age of the trees (Ma et al. IPCC et al., 2013). We concluded that a few deep root observed in our study might play an important role in improving locust tree resistance to drought and sustaining tree growth. Our sampling was an integration of annual growth that encompassed the water availability of the year given the limited infiltration of the periodic rainfall events. As described above, rainfall infiltration depth was no more than 2m (Liu et al., 2010), deep soil moisture (below 2m) would not affected by the periodic rainfall, i.e., the wettest part of the year versus both wet and dry period, it could reflect the effect of long-term meteorological factors on local area, rather than the short-term ones. Ma et al. (2012) also showed that the root density decreased with the horizontal distance from the tree trunk. Thus, the coring points selected in our study in the same position relative to the tree trunk might have avoided differences in the horizontal root distribution. The specific sampling positions might have influenced the shallow root distribution more than that of the deep roots. Therefore, comparing the deep root distribution at the same horizontal sampling position in different sites was a suitable approach.

The rooting depth is recognized as a key trait that underlies the resistance or resilience of trees under drought events (Nardini and Valentino, 2016). Our findings suggest that the vertical extension of the rooting depth and tree height might be key functional traits that determine plant growth in drought-affected areas. We found that the rooting depth of locust trees was positively correlated with the mean annual rainfall, but further studies of the shifts in the belowground root traits (e.g., root xylem size) and aboveground functional traits (e.g., leaf area) are needed to advance our understanding of the roles of the roots in the response to drought (Choat et al., 2018). Nevo (2012) suggested that differences in the slope aspect may be suitable for assessing the mechanisms that allow plants to adapt to different environments under climate change. Considering both the aboveground and belowground effects will be critical for predicting future changes in forest ecosystem function (Collins et al., 2020). Our results provide useful insights into how environmental factors might control the belowground and aboveground functional traits of plants.

## Data Availability Statement

The original contributions presented in the study are included in the article/supplementary material, and further inquiries can be directed to the corresponding author.

## Author Contributions

ML and LX were responsible for manuscript drafting and editing. WY was contributed to experimental design and analysis. YJ was contributed to field sampling and root cleaning. CJ was contributed to Interprate the data of work, and revise our manuscript critically. All authors contributed to the article and approved the submitted version.

## Funding

This study was supported by the National Science Foundation of China (project number 41671510) and the Key Program of Research and Development in Shaanxi Province (project number 2020NY-155).

## Conflict of Interest

The authors declare that the research was conducted in the absence of any commercial or financial relationships that could be construed as a potential conflict of interest.

## Publisher’s Note

All claims expressed in this article are solely those of the authors and do not necessarily represent those of their affiliated organizations, or those of the publisher, the editors and the reviewers. Any product that may be evaluated in this article, or claim that may be made by its manufacturer, is not guaranteed or endorsed by the publisher.
